# An Observational Study Evaluating the Introduction of a Prolonged-Release Protein Substitute to the Dietary Management of Children with Phenylketonuria

**DOI:** 10.3390/nu12092686

**Published:** 2020-09-03

**Authors:** Anita MacDonald, Catherine Ashmore, Anne Daly, Alex Pinto, Sharon Evans

**Affiliations:** Dietetic Department, Birmingham Women’s and Children’s Hospital, Steelhouse Lane, Birmingham B4 6NH, UK; catherine.ashmore@nhs.net (C.A.); a.daly3@nhs.net (A.D.); alex.pinto@nhs.net (A.P.); evanss21@me.com (S.E.)

**Keywords:** phenylketonuria, diet therapy, phenylalanine, protein substitute, gastrointestinal symptoms, prolonged release

## Abstract

Dietary restriction of phenylalanine combined with a protein substitute prevents intellectual disability in patients with phenylketonuria (PKU). However, current protein substitutes are associated with low adherence owing to unpalatability and burdensome administration regimens. This prospective, observational acceptability study in children with PKU assessed the use of a prolonged-release protein substitute designed with an ethyl cellulose and arginate coating masking the bitter taste, smell and reducing the osmolarity of free amino acids. The study product was mixed with the subject’s food or drink and replaced ≥1 dose per day of the subject’s usual protein substitute for 7 days. Seven of 13 subjects were able to take their prescribed dose over the 7 day period. Most subjects mixed the test protein substitute with food or fruit juice. Reduced blood phenylalanine levels (*n* = 5) and improved phenylalanine/tyrosine ratio (*n* = 4) were recorded from baseline to Day 7, respectively. Four subjects reported fewer gastrointestinal symptoms compared to baseline. There were no cases of diarrhoea, constipation, bloating, nausea or vomiting. No adverse reactions were reported. In conclusion, the novel prolonged-release protein substitute was taken in a different way to a typical protein substitute and enabled satisfactory blood phenylalanine control. The study product was well tolerated; subjects experienced fewer gastrointestinal symptoms than with their previous treatment. Although the results of this pilot study provide reassuring data, longer-term studies evaluating adherence and blood phenylalanine control are necessary.

## 1. Introduction

Phenylketonuria (PKU) is a rare metabolic disorder caused by a deficiency of phenylalanine hydroxylase (PAH), the enzyme that catalyses the hydroxylation of phenylalanine to tyrosine, and which leads to irreversible intellectual impairment in untreated children [1]. While there is no cure for PKU, the dietary restriction of phenylalanine has been highly successful in preventing intellectual disability and achieving near-normal intellect. However, current dietary treatments are associated with some major issues, such as low adherence attributed to unpalatable and burdensome dietary supplements and subtle but chronic neuropsychological impairments despite early intervention, particularly in adulthood, including mood and psychiatric issues [2,3,4,5].

Lifelong dietary management of PKU involves severe restriction of phenylalanine plus supplementation with protein substitutes, usually consisting of phenylalanine-free amino acids [6]. Protein substitutes provide essential and nonessential amino acids and commonly include micronutrients that would otherwise be lacking in a low-phenylalanine diet [6,7]. Since the 1960s, when the first manufactured protein substitutes were introduced, improvements have been made to their nutritional composition, presentation, taste and acceptability [7,8]. Currently, protein substitutes are available in a variety of forms, including powders, gels, liquids and tablets [9], and are traditionally administered as high-volume hyperosmolar drinks (if diluted with too little water, they may cause abdominal pain, diarrhoea or constipation [10]).

Generally, many patients have a poor acceptance of protein substitutes and parents struggle to ensure that their children take them as prescribed [11,12]. Poor adherence to a low-phenylalanine diet and protein substitute increases with age and is associated with worsening of blood phenylalanine control [11,13,14]. Furthermore, children may take up to an hour to take their full dose of protein substitute, with some failing to take the prescribed quantity [15]. Although reasons for poor adherence to protein substitutes are multiple, the bitter taste and aftertaste of synthetic amino acids are frequently reported as important factors [9,16]. Manufacturers have tried to improve the taste by lowering the quantities of unpalatable sulphurous and dicarboxylic amino acids and adding flavourings and sweeteners, but the results have been suboptimal [17], with minimal impact on aftertaste [18]. Commonly, children complain of breath odour following consumption of protein substitutes [19]. Although reports of adding protein substitute to food are few [20], this is perceived to have low acceptance [21].

Synthetic amino acids bypass degradation by proteases in the digestive process, resulting in blood levels that are higher, peak faster and decrease more quickly than when compared with natural protein. Therefore, it is recommended to take synthetic protein substitutes in small, frequent dosages in equally distributed amounts [8]. If protein substitutes are taken less frequently, there may be wide variations in blood phenylalanine levels over 24 h, which is associated with reduced blood phenylalanine control in PKU [8,22]. Protein substitutes with the ability to delay absorption of phenylalanine and tyrosine, mimicking physiological absorption kinetics, are expected to improve the rate of protein accretion, minimizing fluctuations in quantitative plasma amino-acid levels [8]. However, despite previous efforts, there has been little success in developing a slow-release protein substitute that mirrors the physiological absorption kinetics of intact natural proteins.

There is a need for more palatable and physiological protein substitutes that are both effective at reducing 24 h variability in phenylalanine levels and are accepted by patients. Here, we report outcomes from an observational study assessing the introduction of a prolonged-release protein substitute to the diets of children with PKU [16,23].

## 2. Materials and Methods

### 2.1. Study Design

This was a prospective, observational acceptability study performed in children with PKU aged 3–16 years who attended a single clinic at Birmingham Women’s and Children’s Hospital, Birmingham, United Kingdom. Caregivers of eligible children were identified and sent a study information sheet. Research dietitians discussed the study details with interested parents and patients on their request. This study was conducted according to the requirements stated by the UK Advisory Committee for Borderline Substances (ACBS). This is a committee that recommends dietary products to be reimbursed by the National Health Service (NHS). The study product met the criteria of a Type 2 product: *“a formulation which was broadly similar in composition to existing products already on the market.”* According to ACBS guidelines, *“All acceptability studies must be for at least 1 week and at least 15 patients must complete the study. Where nutritional products are intended for use in very rare conditions, such as inherited metabolic disorders, fewer patients are acceptable*”. Thirteen patients were enrolled for 7 days of treatment and were able to complete the study, but only 54% (7 of 13) were able to take 100% of the prescribed product and were included in the final analyses. These studies conformed to the principles of good clinical practice.

### 2.2. Inclusion and Exclusion Criteria

Inclusion criteria included: male or female; aged 3–16 years; proven diagnosis of PKU requiring a protein substitute; already taking a protein substitute; and willing to take the study product for 7 days.

Exclusion criteria included: presence of serious concurrent illness; chief investigator’s uncertainty about the willingness or ability of the patient to comply with protocol requirements; participation in any other study involving investigational or marketed products within two weeks prior to study entry; and children who received antibiotics over the two weeks prior to the study.

### 2.3. Study Product

The study product, PKU GOLIKE PLUS 3–16, (APR Applied Pharma Research, Switzerland) is a protein substitute for oral use in the form of off-white/light yellow granules, consisting of a prolonged-release amino-acid mixture with vitamins and minerals and other nutrients (i.e., carnitine, taurine, choline and inositol). The study product was developed with a coating able to overcome practical issues associated with free amino acids, such as bitter taste, smell, aftertaste and osmolarity. The coating consisted of ethyl cellulose plus alginates encasing granules of amino acids (without phenylalanine). The study product could be mixed with food or fruit juice or taken as granules. It was gluten and lactose-free and contained no added fat, with a nutritional profile suitable for patients aged 3–16 years (Table 1).

The study product was introduced into the standard therapeutic diets of enrolled children for 7 days by replacing at least one dose per day of the patient’s usual protein substitute, according to ACBS requirements. Considering the short study duration, all the protein substitute requirements were not replaced as patients had little time to adapt to a different type of protein substitute given in another format.

Patients followed their usual low-phenylalanine diets during the study. Any foods containing protein up to 0.5 g/100 g and fruits and vegetables containing phenylalanine up to 75 mg/100 g were given without measurement. We aimed to maintain the same total protein equivalent intake for each patient whilst taking the prolonged-release protein substitute.

### 2.4. Preparation of Study Product

The research dietitians explained the study product’s characteristics and its theoretical advantages to the caregivers and patients. Verbal and written information was given about how to administer the study product, i.e., granules could be taken either in liquids or semisolid foods with a creamy consistency (fruit smoothies, low-protein vegetable soup, fruit or vegetable purees, low-protein puddings or desserts). Each caregiver and patient were also given a practical demonstration on how to mix the study product. The type of food or drink that the study product was mixed with was selected by the patient.

### 2.5. Assessments

Subject demographics were recorded at study baseline. Prior to treatment initiation, information was recorded regarding the current protein substitute (dose, type, palatability and presence of any gastrointestinal side effects).

During treatment, parents/caregivers and subjects completed daily questionnaires to record treatment preparation/administration and any problems, including adverse events or gastrointestinal side effects. An additional questionnaire was completed at the end of treatment.

All patients had known adherence with their usual protein substitute prior to entering this study as well as routine blood spot phenylalanine monitoring, with three retrospective blood spots available in addition to blood tests taken during the study.

A fasting blood spot for phenylalanine was taken at home by parents/caregivers both before and at the end of the treatment period. Children aged ≤12 years aimed to maintain blood phenylalanine between 120–360 µmol/L; children aged >12 years aimed to maintain levels between 120–600 µmol/L. Early-morning fasted blood spots were collected on filter cards (Perkin Elmer 226, Greenville, SC, USA, UK Standard NBS). Blood specimens were sent via first-class post to the laboratory at Birmingham Children’s Hospital. All cards had a standard thickness and the blood phenylalanine concentrations were calculated on a 3.2 mm punch by Waters Xevo TQD tandem mass spectrometer (Elstree, Herts, UK).

### 2.6. Ethical Permission

The Solihull Research Ethics Committee granted a favourable ethical opinion (REC reference: 19/WM/0151, IRAS project ID: 256519). Written consent was obtained for all subjects from at least one caregiver with parental responsibility and written assent obtained from the subject if appropriate for their age and level of understanding.

### 2.7. Statistical Analysis

Descriptive statistics were used to examine demographics and disorder characteristics, protein substitute use, adverse events and plasma phenylalanine and tyrosine levels.

## 3. Results

### 3.1. Subjects

A total of 13 subjects were enrolled into the acceptability study (12 with classical PKU; 1 with moderate PKU); mean age was 11.6 years (range 7 to 16 years). All subjects were diagnosed with PKU via newborn screening and started a low-phenylalanine diet from the time of screening. None were treated with sapropterin as an adjunctive therapy. At the start of the study, the subjects routinely took either one or two different types/brands of protein substitute daily (eight subjects took a single protein substitute daily; five subjects took two different types). Seven of the subjects routinely took a protein substitute derived from casein glycomacropeptide (CGMP), and three subjects took this as their sole source of protein substitute, with four subjects taking CGMP with an amino acid substitute. In total, five subjects usually took both a liquid and a powdered protein substitute, five took a liquid substitute only, and three took a powdered substitute only. The median (range) dose of protein equivalent from protein substitute was 1.4 (1.0–3.1) g/kg/day.

### 3.2. Substudy Cohort Results

The substudy cohort consisted of subjects who were able to take the entire prescribed dose of the study product (*n* = 7; mean age 10.9 years) (Table 2). The mean percentage of the prescribed dose consumed over 7 days for these patients was 98% (range 92–100%). Subjects that consumed less than 90% of the prescribed dose during the 7 days of treatment are not included in the substudy cohort analyses. Subjects were prescribed either 15 g/day (*n* = 6) or 20 g/day (*n* = 1) protein equivalent of the study product to replace one dose of the total protein substitute intake. The study product provided 20–27.3% of the subject’s usual protein equivalent intake per day; the remaining protein equivalent intake was from each subjects’ usual protein substitute. Three subjects (Subjects 1, 2 and 6) were 5 g of protein equivalent short of their pre-study dose, due to differences in sizes of protein substitute sachets/pouches.

### 3.3. Administration of Study Product

The study product was mixed with a variety of different drinks and foods, but the preferred method was to prepare it as a fruit smoothie or mixed with fruit juice (Table 2). All subjects or their parents found the study product easy to prepare. Seven of 13 children commented that the product had little taste or that it tasted “okay”. The reason why children (*n* = 6 of 13) were unable to take the entire prescribed dose of the study product was primarily because of texture (described as bitty and gritty) and one child described the amount of powder as “a little overpowering” when added to food.

### 3.4. Adherence with Study Product

All but one of the subjects included in the substudy cohort took 100% of the prescribed study product on at least one day during the study period; five subjects took 100% of the dose every day. The lowest percentage daily intake of the study product was 75%, which occurred once throughout the study (Day 1, Subject 2).

### 3.5. Blood Phenylalanine and Tyrosine Control

**Figure 1:**
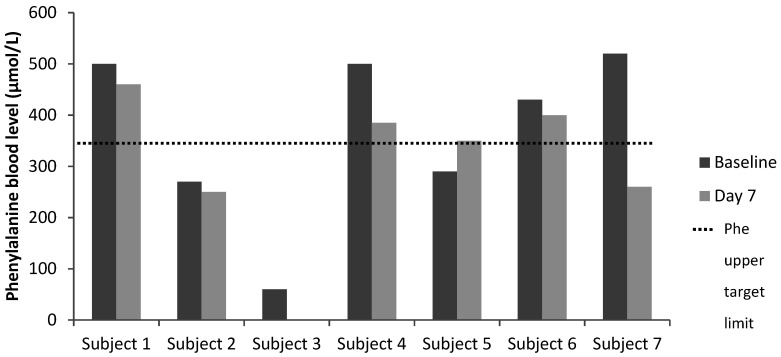
Phenylalanine blood levels of the substudy patient cohort over the study period. Phe target range was 120–360 µmol/L for patients ≤12 years and 120–600 µmol/L for patients >12 years. (Note: Sample labelling issue for Subject 3 on Day 7, so no result recorded). Abbreviation: Phe: phenylalanine.

Blood phenylalanine and tyrosine control was satisfactory over the study period, with lower blood phenylalanine levels recorded in five of the seven subjects (Subject 1: −40%, Subject 2: −7%, Subject 3: NA, Subject 4: −33%, Subject 6: −7%, Subject 7: −50%) (Table 3 and Figure 1). One subject had blood sample labelling issues, so blood phenylalanine was not reported whilst on the study product (Subject 3). The blood phenylalanine level increased in one child (Subject 5: +21%) but remained within the recommended target range. Tyrosine baseline and Day 7 data were available for five subjects: tyrosine levels increased in three subjects and were lower in two subjects. The phenylalanine/tyrosine ratio improved in four of five subjects.

### 3.6. Gastrointestinal Symptoms

The study product was well tolerated by all seven subjects for the entire study period. Four subjects reported fewer gastrointestinal symptoms, with less burping, flatulence and regurgitation whilst taking the study product. In one subject, ‘severe’ burping, flatulence and regurgitation was recorded at baseline and reduced to ‘mild’ with the study product. There were no cases of mild, moderate or severe diarrhoea, constipation, bloating/distension, nausea or vomiting during the study. There was one case of moderate abdominal discomfort and pain (attributed to menstruation).

### 3.7. Adverse Events

No adverse reactions were reported.

## 4. Discussion

In this study, we assessed the introduction of a novel protein substitute with a coating that supports a more physiological release of amino acids than existing substitutes and also masks their bitter taste, aftertaste and smell [16]. The study design followed the guidelines of the UK ACBS with the objective of evaluating the acceptability of the new protein substitute with a limited number of subjects and within a short evaluation time. Despite a short time period for adaptation to the study product, subjects did not report that taste and smell were an issue. During the study, most of the subjects either showed improved blood phenylalanine and tyrosine control or at least maintained them within their target range. This is an encouraging preliminary outcome.

Many patients with PKU are food neophobic [24,25]: and are very suspicious of anything different. Consequently, this short evaluation study was particularly challenging. The study product was dissimilar from current protein substitutes, which patients were well used to taking. Each child understood the potential advantages of taking a prolonged-release protein in terms of impact on blood phenylalanine control and product taste and was very open to trying this new product. However, none had previously experienced taking a protein substitute added to their usual food or drink, so this was an unfamiliar concept to them. The abrupt introduction of a new substitute was overwhelming to some children. Understandably, it is likely to take time to adapt to change, and a slower, more gradual introduction may have been more acceptable. Thereby, a program of slow and systematic introduction, with consistent support given by the PKU team, is essential. Additionally, accompanying educational messages may significantly influence motivation and persistence when introducing any new protein substitute [26]. Generally, patients with PKU and their caregivers welcome new treatments, changes in treatment strategies, new foods, and different presentations of protein substitutes if it improves acceptability and tolerance of treatment [27].

Most of the subjects added the study product to fruit smoothies or juice. This form of administration was quick and similar to their current method of taking protein substitute. This study group has always treated protein substitute differently to food, consumed it immediately before or after meals. If the study evaluation had been extended, they might have eventually accepted mixing the new protein substitute with food or drinks. Due to the study product’s coating, the taste and smell were masked, enabling the protein substitute to be taken as an ‘add-on’ to meals and snacks without affecting the taste of the original food. This type of protein substitute may be particularly useful for patients returning to a low-phenylalanine diet, who have unpleasant memories associated with the smell and taste of protein substitute. It may also be helpful during pregnancy for women struggling with nausea and vomiting associated with the taste and smell of protein substitutes [28]. The excipients used for the coating of the study product are considered safe for pregnancy and lactation [29].

The study product was well tolerated over the treatment period. Protein substitutes are known to have a high osmolarity and therefore may cause gastrointestinal upsets, including abdominal pain, diarrhoea and constipation [12,15,30]. In this cohort, there were no cases of diarrhoea, constipation, bloating/distension, nausea or vomiting during the study. Four subjects reported improvements in the severity of burping, flatulence and regurgitation that they had experienced while receiving their previous treatment regimen. Importantly, there were no adverse reactions reported during the study period. The use of an ethyl cellulose and alginate coating on the study product resulted in granules of amino acids that were stable in the stomach with a gradual disintegration during small-intestine transit [29,31]. This may explain the favourable gastrointestinal tolerability in this study. Both excipients (ethyl cellulose and alginate) are widely used in pharmaceutical technology and recognised as safe for use in medical foods [16]. It would be important to observe whether benefits to gastrointestinal tolerance could be observed longer-term, particularly if the study product provided a higher proportion of protein-substitute intake.

In recent years, several new protein-substitute compositions and formulations have been developed with the aim of improving adherence, which remains suboptimal especially in adolescents and adults with PKU [32]. However, all contain free L-amino acids with absorption kinetics that are more rapid than intact protein sources, causing a lower biological and functional efficacy [33,34,35]. The introduction of a prolonged-release protein substitute with the ability to prolong absorption of synthetic amino acids is expected to minimise blood phenylalanine level fluctuations and improve phenylalanine control and other metabolic markers in PKU [8,23], but longer-term studies are required to examine the impact on blood phenylalanine control and 24 h blood phenylalanine variability.

There were limitations to this study evaluation. It represented a small patient population with a limited treatment period. Only around 20 to 30% of the usual protein substitute requirements were substituted with the study product due to the short time of adaptation. There were limited blood phenylalanine control data. Additional, more extensive studies performed in a larger population and over a longer timeframe will provide more evidence regarding the adherence and tolerability of this protein substitute, together with impact on metabolic control in patients with PKU.

## 5. Conclusions

In children with PKU, partial replacement of standard protein-substitute with a prolonged-release protein substitute was achievable. The study product was mixed with food or fruit juice. Subjects maintained satisfactory blood phenylalanine control and were able to take their protein substitute in a different way to their usual practice. The prolonged-release protein substitute was well tolerated, with subjects experiencing fewer gastrointestinal symptoms than with their previous treatment regimen.

## Figures and Tables

**Table 1:** Nutritional declaration for the study product (PKU GOLIKE PLUS 3–16) ^1^.

Component	Per 100 g	Per Sachet of 24 g
Energy	280 kcal/1187 kJ	67 kcal/286 kJ
Fat	0 g	0 g
of which saturated	0 g	0 g
Carbohydrate	4.3 g	1.0 g
of which sugars	0 g	0 g
Fibre	7.1 g	1.7 g
Protein equivalent ^1^	62.2 g	15 g
Salt	0.06 g	0.015 g
**Amino Acids**		
L-serine	2.5 g	0.6 g
L-threonine	3.8 g	0.9 g
L-leucine	8.6 g	2.1 g
Glycine	3.8 g	0.9 g
L-alanine	2.3 g	0.5 g
L-arginine	3.0 g	0.7 g
L-cysteine	1.5 g	0.4 g
L-glutamine	15.0 g	3.6 g
L-histidine	2.1 g	0.5 g
L-aspartic acid	4.5 g	1.1 g
L-proline	4.5 g	1.1 g
L-isoleucine	4.1 g	1.0 g
L-lysine	5.3 g	1.3 g
L-tryptophan	1.5 g	0.4 g
L-valine	3.8 g	0.9 g
L-methionine	1.0 g	0.3 g
L-tyrosine	7.5 g	1.8 g
**Vitamins**		
Vitamin A (RE)	1295 mcg	311 mcg
Vitamin D	25 mcg	6.0 mcg
Vitamin E (αTE)	13 mg	3.2 mg
Vitamin K	100 mcg	24 mcg
Vitamin C	135 mg	32.31 mg
Thiamine	2.0 mg	0.5 mg
Riboflavin	1.9 mg	0.5 mg
Niacin	27 mg	6.4 mg
Vitamin B6	2.6 mg	0.6 mg
Folic acid	267 mcg	64.1 mcg
Vitamin B12	4.2 mcg	1.0 mcg
Biotin	54 mcg	13 mcg
Pantothenic acid	11 mg	2.6 mg
**Minerals**		
Potassium	1250 mg	300 mg
Calcium	1339 mg	321 mg
Magnesium	304 mg	72.9 mg
Phosphorus	1060 mg	254 mg
Chloride	0.75 mg	0.18 mg
Sodium	25 mg	5.9 mg
Iron	23 mg	5.6 mg
Zinc	14 mg	3.4 mg
Copper	1.4 mg	0.3 mg
Manganese	2.5 mg	0.6 mg
Selenium	58 mcg	14 mcg
Chromium	46 mcg	11 mcg
Molybdenum	88 mcg	21 mcg
Iodine	225 mcg	54.0 mcg
**Other Nutrients**		
Carnitine	0.08 g	0.02 g
Taurine	0.21 g	0.05 g
Choline	321 mg	77.1 mg
Inositol	214 mg	51.4 mg

^1^ 1 g of protein equivalent = 1.2 g of amino acids. The protein content is provided by the amino acids. Ingredients: L-glutamine, L-leucine, L-tyrosine, L-lysine acetate, glazing agent: ethyl cellulose; calcium hydrogen phosphate dihydrate, maltodextrin, L-aspartic acid, L-proline, L-isoleucine, L-threonine, glycine, L-valine, potassium bicarbonate, L-arginine, L-serine, L-alanine, L-histidine, L-cysteine, L-tryptophan, L-methionine, choline bitartrate, magnesium oxide, iron, maize starch, ferric pyrophosphate, glazing agent: sunflower lecithin, stabiliser: sodium alginate; inositol, taurine, L-ascorbic acid, L-carnitine, zinc sulphate, nicotinamide, DL-alpha tocopheryl acetate, chromium chloride hexahydrate, sodium molybdate, manganese gluconate, calcium-d-pantothenate, cupric gluconate, retinyl palmitate, pyridoxine hydrochloride, thiamine hydrochloride, riboflavin, cholecalciferol, folic acid, potassium iodide, phytomenadione, sodium selenite, D-biotin, cyanocobalamin.

**Table 2:** Baseline demographics and treatment in the substudy cohort.

**Baseline Demographics**							
**Subject**	1	2	3	4	5	6	7
**Age (years)**	11	11	12	9	7	11	15
**Weight (kg)**	60.3	53.3	45.9	25.8	26.6	45.6	55.8
**Height (cm)**	152.3	147.5	155.8	124.5	119.7	154.8	174.4
**PKU classification**	Classical	Classical	Moderate	Classical	Classical	Classical	Classical
**Blood Phe on diagnosis (µmol/L)**	1700	1680	900	1390	1590	2520	2690
**Gender**	Female	Female	Male	Male	Male	Male	Male
**Ethnicity**	Pakistani	Pakistani	White European	White British	Mixed race	White British	White British
**Diet and Protein-Substitute Profile**							
**Natural protein allowance (g/day)**	4.0	4.0	7.0	3.0	6.5	7.5	18.0
**Protein equivalent from usual protein substitutes g/day (g/kg/day)**	60.0 (1.0)	60.0 (1.1)	60.0 (1.3)	80.0 (3.1)	60.0 (2.3)	80.0 (1.7)	60.0 (1.2)
**Number of different protein substitutes/day**	2	2	2	1	2	2	1
**Number of doses/day**	3	3	4	4	4	4	3
**Study Product Treatment Schedule and Preparation**							
**Daily dose (g)**	24.0	24.0	24.0	32.0	24.0	24.0	24.0
**Protein equivalent from study product (g)**	15.0	15.0	15.0	20.0	15.0	15.0	15.0
**Total daily protein equivalent from all protein substitutes (g) ^1^**	55.0	55.0	55.0	80.0	60.0	75.0	55.0
**Protein equivalent from study product** **(% of daily intake)**	27.3	27.3	27.3	25.0	25.0	20.0	27.3
**Method of administration**	In fruit juice	In fruit juice	Food and drinks	In fruit juice	In fruit juice and food	Fruit smoothie	Smoothie
**Timing of administration of study product**	Evening	Evening	Evening	Evening	Morning midday and bedtime	Morning or evening	Morning or evening
**Comments on** **study product**	Left some bits behind on cup	Last bit was hard to take	No comments	No comments	No comments	Required blender to mix	No comments

^1^ Subject 1, Subject 2 and Subject 6 were 5 g of protein short of their pre-study dose due to differences in sizes of protein substitute sachets/pouches when incorporating the study product with existing protein-substitute diet plan. Abbreviations: Phe: phenylalanine; PKU: phenylketonuria.

**Table 3:** Phenylalanine and tyrosine blood levels of the substudy patient cohort during the study.

Subject	1	2	3	4	5	6	7
**Target Phe levels (µmol/L)**	120–360	120–360	120–360	120–360	120–360	120–360	120–600
**Phenylalanine Levels (µmol/L)**							
**Baseline**	500	270	60	500	290	430	520
**Day 7**	460	250	NA ^1^	385	350	400	260
**Tyrosine Levels (µmol/L)**							
**Baseline**	110	120	50	30	60	50	70
**Day 7**	150	160	NA ^1^	NA ^1^	40	60	50
**Phenylalanine/Tyrosine Ratio**							
**Baseline**	4.5	2.3	1.2	16.7	4.8	8.6	7.4
**Day 7**	3.1	1.6	NC ^1^	NC ^1^	8.8	6.7	5.2

^1^ Sample labelling issue. NA: not available; NC: not calculable.
